# The Fog of War: Russia’s War on Ukraine, European Defence Spending and Military Capabilities

**DOI:** 10.1007/s10272-022-1051-8

**Published:** 2022-06-07

**Authors:** Daniel Fiott

**Affiliations:** Justus Lipsius Building, EU Institute for Security Studies (EUISS), 175, rue de la Loi, 00-FG-14, 1048 Brussels, Belgium

**Keywords:** O33, F50, N44

## Abstract

Some … may look at the depleted nature of Russia’s military after its invasion of Ukraine and incorrectly surmise that defence spending increases are not that necessary after all.

When war hits, some degree of analytical humility is required. No one knows how – or when - Russia's war on Ukraine will end and the effects on European security over the medium to longer term (i.e. the next five to ten years) are unknown. Despite the analytical fog that shrouds Russia's war, discussions increasingly focus on how European countries should support Ukraine, how Europe and the United States should view and engage with Russia during and after the conflict and to what extent Europe should bolster its own defences. Such questions have already found material form. Think of the wide-scale provision of weapons and lethal equipment to Ukraine, the enhanced military presence on the European Union's and NATO's eastern flank, the announcements of increased defence spending in Europe, the sanctions designed to disable the Kremlin's war machine or the calls for Ukraine to be speedily brought into the EU. Finland and Sweden have also formally announced their intention to join NATO in response to Russia's actions.

So far, Europeans have shown a high degree of unity in the face of Russia's war – forces have been deployed to the eastern flank and, despite the difficulties on agreeing to an oil and gas embargo on Russia, political unity has ensured that heavy sanctions are in place. However, this unity is likely to be challenged over the coming months and years by a fundamental question that has so far not received sustained and serious attention: How should Europe view and interact with Russia after its invasion of Ukraine? Some elites may desire a détente with Putin, especially with economic interests at stake. Others, however, reject any notion of a diplomatic settlement with Russia and instead call for preparations for a long-term political standoff with the Kremlin. How Europe politically adapts itself to the Russian threat in the coming months and years will be a test of its credibility, unity and autonomy.

Indeed, Russia's war on Ukraine is already giving rise to questions that will have long-term implications for European security, including: To what extent should European countries increase their defence expenditure? How should Europeans invest any additional financial resources for defence? To address such questions, this article looks at some of the implications of Russia's war on Ukraine for European security, and it focuses on some of the direct policy choices that European countries presently face and those that may appear on the horizon. To this end, the paper explores how defence expenditure in Europe may evolve after Russia's invasion and how defence investments may be shaped in the coming years. More specifically, the article looks at the interplay between geostrategic and investment choices.

## Money for nothing?

One of the obvious effects of Russia's February 2022 invasion of Ukraine has been several announcements that European countries will increase their defence expenditures. To be sure, the need to increase military budgets was already a recognised political ambition in Europe; and ever since Russia's initial invasion of Ukraine in 2014, EU and NATO states have been working – however slowly – to increase defence spending. Indeed, the European Defence Agency (2020b) estimates that EU members invested €198 billion in defence in 2020, but only after they collectively cut spending from 2008 to 2014 by €24 billion in the wake of the 2007 financial crash. An important moment in the history of European defence spending occurred in 2014, as the NATO Defence Investment Pledge endorsed in that year called for Allies to meet the 2% of GDP spending guideline. Russia's actions – rather than the divisive rhetoric of former US President Trump – have led a handful of European countries to expand defence spending since 2014.

Russia's further military invasion of Ukraine in 2022 is only likely to lead to more announcements about defence spending hikes in Europe. For example, Germany recently announced that it would substantially increase defence spending and Chancellor Scholz has promised an injection of €100 billion through a "special fund" that will be invested from 2022. Additionally, Berlin has announced that it will ensure that it meets NATO's 2% pledge sooner rather than later. Indeed, this about-face on spending has been viewed as part of a wider paradigm shift in German strategic thinking – what is being called a *Zeitenwende*. Whether this will really occur remains to be seen, but other European states have also pledged to boost their military expenditures. Countries such as France, Italy, the Netherlands, Portugal, Spain and Sweden have proclaimed their intention to spend more in line with NATO and EU targets, and countries that already meet the 2% target – such as several Baltic states and eastern NATO allies – will spend more in future (Arteaga et al., 2022).

Of course, declarations of increased defence spending should be questioned, and it is unclear how fast and to what extent European governments will increase their spending levels in real terms. For example, some analysts have already questioned whether Germany's injection of €100 billion will be enough to close the gap on its 2% of GDP commitment. Such analyses point to how quickly this €100 billion will be swallowed up by the need to replenish depleted munitions stocks, procure heavy transport helicopters, invest in air defence systems and develop future European capability programmes such as the Future Combat Air System (FCAS) and Main Ground Combat System (MCGS) (Giegerich and Schreer, 2022). Other European defence budget increases should also be scrutinised for how far they realistically add new money for defence (rather than simply topping budgets to redress post-2008 budgetary cuts) or whether they will outpace inflation.

Keep in mind that inflation is high today, not least because of inflated energy prices and the COVID-19 recovery. Yet even in 2021, higher inflation conspired to cut global defence spending in real terms by 1.8% (McGerty, 2022). Even if the war in Ukraine has justified announcements of defence spending hikes, the ramifications of the war may create financial pressures that European governments will not find sustainable. As the war drags on, and the economic effects hit the global economy, governments will have to address the effects of the "cost of living" crisis across Europe. Some governments may hold to the idea that increased defence spending is necessary regardless of the economic context, but others may be tempted to invest only incrementally in defence if other public services require enhanced spending. Some may look at the depleted nature of Russia's military after its invasion of Ukraine and incorrectly surmise that defence spending increases are not that necessary after all.

Even in times of major economic upheaval, there is every reason to believe that European governments will enhance their defence spending. Not only do Europe's armed forces drastically need modernisation, but Europe's strategic interests include and go beyond the war in Ukraine. If, as governments have acknowledged, Europe is entering an era of strategic competition where the continent may have to act without the United States, then investment in military capabilities is of paramount importance. As part of this rationale for meeting the challenge of strategic competition vis-à-vis Russia, China and others, Europe will need to invest in its technological, scientific and industrial base in order to ensure that it has the military equipment needed to defend its interests. Such investments may come at the expense of higher debt, so this is really a test of whether Europe acts according to a strategic rather than an economic rationale.

Assuming that there is a higher investment in defence in Europe, the next point to address is how this additional money will be spent. As the German case indicates, some of the investment will be needed to cover essential capability gaps. In this respect, we should not expect these national funds to be exclusively invested in European collaborative solutions (e.g. Germany's insistence on purchasing the US-made F-35 fighter aircraft is conditioned by its nuclear commitments in NATO and the lack of a credible alternative European nuclear delivery air system). What is more, it is unrealistic to expect governments to increase national defence spending without wanting a large proportion of this money to benefit national producers. In this respect, it is worth questioning whether the war in Ukraine will incentivise European governments to develop or procure capabilities together rather than nationally.

## Collaborative defence procurement in the EU

While military capability collaboration does not always reduce project costs overall, there is evidence that the individual contributions to multinational projects can be better managed from a cost perspective. Pushing for European capability solutions, if managed properly, can also lead to greater cross-border cooperation and the enlargement of supply chains (which, in turn, can help increase security of supply). In some cases, major capability projects cannot be handled at a purely national level because of the technologies involved and labour skills required, among other things. In this respect, the direct and quick return on national investment is less obvious but still fruitful if it leads to broader European defence innovation and trustworthy supply chains that cannot be compromised by Europe's strategic rivals. Indeed, we can already observe evidence of this impulse in the work on the FCAS and MCGS projects and EU-level initiatives such as Permanent Structured Cooperation and the European Defence Fund are geared towards further incentivising European cooperation.

The truth is that it is not the norm for European countries to invest national defence budgets into collaborative projects. Indeed, recent data shows that the level of investment in European collaborative equipment procurement is on the decline – one study shows that in 2019 collaborative spending decreased by 13% to low levels not seen since 2005 (European Defence Agency, 2020b, 10). Despite this fact, there has been growing interest in collaborative investment bodies like the EU and NATO. In 2021, 17 out of 30 NATO members signed on to a declaration of intent to create the NATO Innovation Fund. The Fund will be endowed with €1 billion for at least the next 15 years and it will, through the Defence Innovation Accelerator for the North Atlantic, seek to promote and accelerate early-stage defence innovation. The 2022 NATO Madrid Summit will initiate these allied endeavours and the hope is that investments can begin in 2023 (NATO, 2022).

More substantially, the EU has already developed the European Defence Fund (EDF) for collaborative investments in defence innovation and capability prototyping. The EDF is endowed with €8 billion up to 2027, and discussions will begin in 2024 for an additional financial envelop for the period 2028-2035. The EDF is already investing in defence research and capability projects, and all 27 EU member states have the potential to access the Fund for collaborative programmes. It should be noted that one of the precursor programmes to the EDF – the European Defence Industrial Development Programme (EDIDP) – has already invested €500 million in 2020 for space, precision strike, naval, air, cyber and drone systems as well as disruptive technologies such as artificial intelligence (European Commission, 2020). The fact that the EDIDP has benefited 420 industrial entities from 26 EU member states in a single year provides evidence that European countries are increasingly looking to cooperate in the industrial sphere.

Yet the story for the EU does not end here. Indeed, in the wake of Russia's invasion of Ukraine, European leaders met at Versailles on 10-11 March 2022 to discuss how best to respond to the Kremlin. An important element of the Versailles Declaration was the leaders' call for greater investments to fill the critical military capability gaps of Europe's armed forces. Spurred on by the seismic announcement of an additional €100 billion for defence in Germany, and drawing on the experiences of NextGenerationEU, the Declaration asked the European Commission to analyse the defence investment gaps facing the EU and to make recommendations on how to further strengthen the European Defence Technological and industrial Base (European Council, 2022). While we are yet to see how ambitious the Commission will be, or whether EU member states will underwrite further capital borrowing powers for the EU's executive arm, one of the theories is that the EU may start borrowing billions more for investment in the defence sector. Anything close to the €100 billion announced by Germany would be potentially game-changing for European collaborative capability development and open the door for ambitious EU joint procurement and defence planning.

### Investing wisely?

Naturally, any discussions about increased levels of defence spending will imply a need to reflect on the types of military capabilities Europe should procure in the short (2022-2025), medium (2025-2030) and long term (2030+). Even in a context of renewed war in Europe, this discussion is not as easy as it may first appear. Being able to prioritise the development and/or procurement of military capabilities is an intensely political discussion between states that is ultimately conditioned by geographical, industrial and cultural considerations. For example, one can perhaps agree that European states need to substantially and rapidly increase their stocks of main battle tanks and naval vessels. Tanks would address the necessity of repelling any Russian intervention in Europe, frigates and aircraft carriers would allow Europe to exert maritime power. In a context where financial resources are still limited, and where European states have different strategic priorities, should Europe bet on the tanks or naval vessels? This type of question bedevils defence planners across Europe when any decision about European cooperation is considered.

Russia's war on Ukraine has, of course, only sharpened arguments in favour of military capabilities that can deter Moscow from expanding their armed aggression. In fact, Russia's actions have vindicated the European Defence Agency's assessment – made in November 2020, and thus before the 2022 invasion – that European states should urgently invest in main battle tanks, soldier systems/force protection technology, patrol class surface ships, counter unmanned aerial systems, defence-space capabilities and military mobility. Each of these military capabilities can be of use to European armed forces in deterring further Russian aggression, even if the progress in each of these capability areas has been relatively sluggish. For example, the EU has called for modernised tanks, soldier systems and patrol vessels to be ready within the next decade (European Defence Agency, 2020a), but this hardly seems fast enough given the strategic issues confronting Europe.

Indeed, policymakers have tended to view military capabilities as investments for the future. The war in Ukraine has shown that capability acquisition requires credible industrial capacity and reliable supply chains. The war has also shown that an ability to get off-the-shelf equipment and solutions into the field as soon as possible is of the utmost importance. For example, the Ukrainian military are using Turkish-made remotely piloted Batraktar aerial vehicles that cost a fraction of the price of American or Israeli offerings (Witt, 2022). The US defence firm, Lockheed Martin, has also announced that it will double its production of javelin anti-tank missiles, even if this could take up to two years to fully adjust production cycles and timelines (Jones, 2022).[1] Indeed, one of the major issues facing Europe's defence planners and military leaders is that Europe's industry might be unable to completely deal with the surge in demand for military products. This may mean that European suppliers lose to competitors, which could be negatively perceived by those who were reluctant to increase defence spending in the first place.

Of course, the additional aspect that has emerged since the outbreak of war in Ukraine is how best to balance investments between short-term needs and longer-term programmes. For example, many NATO and EU states are focusing on the need to enhance and replenish their own stocks of ammunition and munitions, especially since many governments have transferred existing stocks to Ukraine. The war has also given rise to discussions about the desire to maintain Soviet-era capabilities in European military inventories. The practice at present is to transfer Soviet-era equipment (e.g. armoured vehicles, guns and tanks) to Ukraine because forces in that country are familiar with how to use them. The flip side of this strategy for European governments is to replace Soviet-made transfers with more modernised Western equipment (e.g. Poland replacing its T-72 tanks with American-made Abrams tanks). Again, this short-term need to replenish and replace military equipment may not necessarily work in favour of Europe's defence industry, even if there are credible strategic explanations to buy from non-European suppliers. This, in turn, only adds pressure to European suppliers seeking to capitalise on increased defence spending.

Beyond this relatively new dynamic in the European defence market, however, there is also a need to consider how the war in Ukraine might challenge capability development assumptions in Europe. One of the obvious changes that could be on the horizon is a shift towards more ambitious European investments in missile defence, air interdiction assets and cyberdefence. The apparent need for such capabilities becomes all the more evident when one considers how NATO's defence posture could evolve on Europe's eastern flank. To date, NATO's Response Force has operated on a rotational basis, and this has placed a premium on ensuring that investments in military mobility can ease the speedy movement of troops and equipment in case of further Russian aggression. However, should NATO opt for a permanent presence in Eastern Europe, this will have broad implications for Europe's defence investments.

Indeed, the first step that would have to be taken is the modernisation – and in some cases complete overhaul – of military bases in Central and Eastern Europe. New or refurbished military bases and barracks would have to be connected by secure and modernised transport links, which is already the objective of the EU's work on military mobility, i.e. seeing financial investments into dual-use transport infrastructure (Fiott, 2022). Yet a more permanent NATO presence would equally imply a need for integrated air and missile defence and investments in cyberdefence. Of course, such investments come with sensitive political considerations. For example, to date the EU has only invested in dual-use transport infrastructure, and it would need to find new ways of financing military installations such as land, naval and air bases. An off-budget EU financing tool in the guise of the European Peace Facility could be a possibility.

Additionally, some European governments may balk at the idea that EU financial resources should be spent on installations that will largely house American forces. The risk here being that the EU is perceived to subsidise – directly or indirectly – the US military and its bases in Europe, which can challenge the idea of EU strategic autonomy and, for select EU states, neutrality. Of course, it is equally possible to claim that EU investments in military installations and air and missile defence would put the Union into the "deterrence game" and drastically increase its relevance in European defence – not least because missile defence will also contribute to keeping civilian urban populations safe. Investments of this nature would also be a serious statement of intent to Europeanise the NATO alliance and ensure that the EU and NATO can effectively work together in an era where the US may eventually re-direct troops and military assets to the Indo-Pacific theatre. Any substantial and permanent repositioning of European armed forces to better protect Europe's eastern flank would also clearly need modern and secure military bases and transport links, regardless of the future US footprint in Europe.

## Conclusions

In the face of Russia's military aggression, European countries are now faced with a number of dilemmas that will seriously challenge its security over the next decade. First, there is a need to address Russia but this will not be easy. Some may argue for a complete strategic downgrading of Moscow, but it is not easy to see how this might be achieved when Russia sits on a formidable nuclear arsenal. Alternatively, while Russia's conventional forces may be bruised following its invasion of Ukraine, and

1 And for a helpful corrective to the title of Witt's (2022) New Yorker piece, see Calcara et al. (2022).

its defence industrial sector severely wounded through sanctions, Vladimir Putin may use the coming years to reinvigorate its position by developing stronger ties with Beijing for technologies, raw materials and services. Furthermore, Russia may use its nuclear forces to probe and challenge Europe's unity and security, while also relying on tools such as cyberattacks, espionage and energy to disrupt European economies and buy time for a reinvigoration of Russian forces. In this regard, the strategic conversation in Europe is likely to revolve around enhanced deterrence and an upgrading of conventional forces.

However, Europe faces an important challenge to its overall standing in international affairs. While the war in Ukraine will consume much of Europe's political and economic bandwidth, it is necessary not to neglect the wider world. If anything, Russia's invasion is already raising questions about global food security and energy prices, and such structural issues – when married to challenges such as China's global power status and climate change – imply that Europe cannot only focus on its eastern flank. Being able to balance Europe's global and regional interests in a context of finite resources will increasingly shape what type of actor Europe will become in global affairs. Despite this serious consideration, the immediate effects of Russia's war in Ukraine will likely raise questions about the extent of increased European defence spending and how best to use these additional resources to modernise and bolster Europe's military capabilities.
